# A Unified Level Set Framework Combining Hybrid Algorithms for Liver and Liver Tumor Segmentation in CT Images

**DOI:** 10.1155/2018/3815346

**Published:** 2018-08-09

**Authors:** Zhou Zheng, Xuechang Zhang, Huafei Xu, Wang Liang, Siming Zheng, Yueding Shi

**Affiliations:** ^1^Institute of Mechanical Engineering, Zhejiang University, Hangzhou 301127, China; ^2^School of Mechanical and Energy Engineering, Ningbo Institute of Technology, Zhejiang University, Ningbo 315100, China; ^3^Department of Minimally Invasive Surgery for Hepatobiliary Hernia, Ningbo Li Hui-Li Hospital, Ningbo 315100, China

## Abstract

Accurate and reliable segmentation of liver tissue and liver tumor is essential for the follow-up of hepatic diagnosis. In this paper, we present a method for liver segmentation and a method for liver tumor segmentation. The two methods are grounded on a novel unified level set method (LSM), which incorporates both region information and edge information to evolve the contour. This level set framework is more resistant to edge leakage than the single-information driven LSMs for liver segmentation and surpasses many other models for liver tumor segmentation. Specifically, for liver segmentation, a hybrid image preprocessing scheme is used first to convert an input CT image into a binary image. Then with manual setting of a few seed points on the obtained binary image, the following region-growing is performed to extract a rough liver region with no leakage. The unified LSM is proposed at last to refine the segmentation result. For liver tumor segmentation, a local intensity clustering based LSM coupled with hidden Markov random field and expectation-maximization (HMRF-EM) algorithm is applied to construct an enhanced edge indicator for the unified LSM. With this development, expected segmentation results can be obtained via the unified LSM, even for complex tumors. The two methods were evaluated with various datasets containing a local hospital dataset, the public datasets SLIVER07, 3Dircadb, and MIDAS via five measures. The proposed liver segmentation method outperformed other previous semiautomatic methods on the SLIVER07 dataset and required less interaction. The proposed liver tumor segmentation method was also competitive with other state-of-the-art methods in both accuracy and efficiency on the 3Dircadb database. Our methods are evaluated to be accurate and efficient, which allows their adoptions in clinical practice.

## 1. Introduction

Segmentation is an image processing operation for identifying an anatomical structure from the surrounding tissues. In the area of computed tomography (CT) based clinical hepatic diagnosis, accurate and reliable segmentation of liver tissue and liver tumor is essential for the follow-up treatment planning and evaluation and computer-aided surgery. In current clinical practice, manual delineation of liver and liver tumor on each slice is still typically performed by radiologists, which could obtain the arguably most accurate segmentation results, but is time-consuming, tedious, and laborious and introduces interobserver variability. Additionally, due to the blurry edges and low level of contrast characterizing the CT images, liver segmentation is regarded as a challenging task. The segmentation of liver tumor encounters the same problem due to the low contrast, ambiguous boundaries, and intensity inhomogeneities. Therefore, the development of sophisticated segmentation algorithms has become a major research focus in medical image computing with the potential to provide accurate, effective, and robust approaches for clinical practice. In the past decade, many remarkable liver and liver tumor segmentation methods have been presented with varying degrees of success. These methods can be roughly classified into two categories: automatic and semiautomatic methods, depending on whether the user interaction is required.

Among automatic liver segmentation algorithms, model-based methods have proved to be the most effective one, where prior anatomical knowledge of the target organ is incorporated into the segmentation process [1]. In MICCAI 2007 liver segmentation challenge [2], the top three algorithms [3–5] for fully automatic liver segmentation are all based on the statistical shape models (SSMs). But SSMs suffer from a major limitation that they tend to overly constrain the shape deformations and overfit the training data due to the small size of training data [6]. To increase the flexibility of SSMs, Zhang et al. [7] proposed a Sparse Shape Composition (SSC) shape prior modeling method to tackle limitations of SSMs in a unified framework. This method was extensively validated on 2D lung localization and 3D liver segmentation and exhibited better performance in both studies than state-of-the-art methods. Inspired by previous work, Shi et al. [6] introduced a novel framework for accurate and robust liver segmentation in portal phase of abdominal CT images based on active shape models (ASMs). The highlight was a new multilevel local region-based SSC (MLR-SSC) to increase the flexibility of shape prior models and capture the detailed local shape information more faithfully. Besides, a mounting interest continues for achieving automatic segmentation via deep-learning techniques [8–12]. Lately, Dou et al. [10] presented a novel and efficient 3D fully convolutional network equipped with a 3D deep supervision mechanism for 3D image segmentation. Lu et al. [11] proposed a 3D liver location and segmentation method with a convolutional neural network (CNN) and graph cuts. Firstly, 3D CNN was employed for liver detection and probabilistic segmentation. Then graph cuts and learned probability map were used to refine the initial segmentation. In [12], Yang et al. introduced a deep image-to-image network which was improved with adversarial training. Their method was trained on annotated data of more than 1000 3D datasets. Validation showed their method can achieve promising segmentation results as well as a faster processing speed.

An increasing number of automatic methods are already available for liver tumor segmentation. For instance, Goetz et al. [13] presented an automatic algorithm, where the liver was required to be segmented first, then the tumor segmentation was achieved by classifying all voxels into healthy or tumorous tissue using Extremely Randomized Trees with an auto-context learning scheme. The highlight was that they applied domain adapted learning from sparse annotations (DALSA) to learn from only sparse annotations and to achieve a fast set-up for new image settings. Moghbel et al. [14] proposed a method, based on a hybrid of fuzzy c-means algorithm with cuckoo optimization and random walkers method with priors. Besides, deep-learning schemes are widely used as a powerful alternative for conventional machine learning due to the great model capacity and the ability to learn highly discriminative features for liver tumors. Li et al. [15] proposed an automatic method with deep CNNs. Their method was compared with three popular machine learning algorithms AdaBoost, Random Forests, and support vector machine to show superiority. In [16], the proposed tumor detection method consisted of two steps. The first step was to segment the liver from the CT images. The second step was to calculate the probability of each pixel in the segmented liver belonging to tumors by the use of a deep CNN. Sun et al. [17] designed a multichannel fully convolutional network (MC-FCN) to segment liver tumors from multiphase contrast-enhanced CT images. This method can make full use of the characteristics of different enhancement phases of CT images, and the results showed it provided greater accuracy and robustness than previous methods.

However, fully automatic methods for liver and liver tumor segmentation, such as aforementioned segmentation approaches, sometimes require massive training datasets, bringing time-consuming training process and statistical model construction. Moreover, they generally suffer from lower accuracy and robustness, as well as a significant higher computational cost [18]. On the contrary, semiautomatic method that allows a fast and accurate segmentation under full user control is competitive and is a key requirement for clinical practice [2]. In the following, we briefly review a few semiautomatic methods, respectively, for liver and liver tumor segmentation.

For semiautomatic liver segmentation, Yang et al. [19] demonstrated a hybrid method, where the CT volume was initially segmented through a customized fast-marching LSM with multiple manually selected seed points and followed by a threshold-based LSM to refine the initial segmentation. Yamaguchi et al. [20] proposed a method based on a correlation map of locoregional histogram and probabilistic atlas. In this method, a liver candidate region was extracted first by the region-growing method and followed by a correlation map and probabilistic atlas to extract the final liver region. Chartrand et al. [21] introduced an algorithm using Laplacian mesh optimization. Firstly, an approximate 3D model of the liver was initialized from a few user-generated contours to globally outline the liver shape. Then the model was automatically deformed by a Laplacian mesh optimization scheme to delineate the liver. Zareei et al. [22] proposed a novel active contour model (ACM) based method, where preprocessing was employed first to extract the initial contour, then a genetic algorithm was applied to obtain optimal parameters for the ACM, which was applied at last to refine the initial contour. Eapen et al. [23] proposed a semisupervised liver segmentation technique in a Bayesian level set framework. In this framework, Bayesian probability model with spatial prior was utilized to initialize the level set and to derive an enhanced variable force and an edge indication function, which helped level set evolution to reach genuine liver boundaries.

For semiautomatic liver tumor segmentation, Häme et al. [18] proposed a method with hidden Markov measure field model and nonparametric distribution estimation. The user was asked to select two points on opposite edges of the tumor for region of interest (ROI) construction before segmentation. And after segmentation, a postprocessing operation was also used to remove the overflow of segmentation. Wu et al. [24] described an improved fuzzy c-means and graph cuts based 3D segmentation method, where a tumor ROI was extracted first through confidence connected region-growing algorithm, then a kernelized FCM with spatial information was incorporated in graph cuts segmentation to achieve the final segmentation. Hoogi et al. [25] proposed an adaptive local window to improve level set for liver tumor segmentation. The window was estimated separately for each point, over iterations of the segmentation process, and for each individual object. This method outperformed other three energy models and showed significantly better segmentation when tackling complex lesions. Conze et al. [26] introduced a novel approach, which applied random forest on supervoxels and involved robust multiphase cluster-wise features extracted from registered multiphase contrast-enhanced CT scans. Method evaluation showed this approach could segment parenchyma, active and necrotic tissues accurately.

One of the reasons that discourage the use of semiautomatic segmentation approaches is that high interaction is required. For instance, among some aforementioned approaches, the type of high interaction applied varies from setting multiple seed points to extensive manual refinement for postprocessing. Moreover, the segmentation accuracy still leaves a room for improvement. Thus, in order to achieve more accurate and efficient segmentation with less interaction, we present a reliable framework for liver and liver tumor segmentation based on a novel unified LSM. In the following, we briefly introduce the LSMs and explain how previous work differs from ours.

Techniques based on LSMs have been extensively investigated for liver and liver tumor segmentation [19, 27–30], because LSMs can handle complex topological changes in a nature and effective way and integrate image information and model properties for optimal segmentation as well. Considering liver segmentation, Suzuki et al. [27] proposed a level set framework for liver segmentation in both CT and MRI images. In this method, a fast-marching LSM with multiple seed points was applied first to roughly determine the liver boundaries and followed by a geodesic-active-contour model coupled with a level set algorithm to refine the initial boundaries. Wang et al. [28] demonstrated an automatic method based on a shape–intensity prior level set combining probabilistic atlas and probability map constraints for liver segmentation. A rough segmentation result was obtained by a maximum a posteriori classification of the probability map, and the final liver segmentation was produced by the evolution of shape–intensity prior level set within the most likely liver region. Li et al. [29] proposed a new fuzzy LSM to facilitate medical image segmentation. This LSM integrated spatial fuzzy clustering and validation results confirmed its effectiveness for liver and liver tumor segmentation as well. Li et al. [30] introduced a level set model incorporating likelihood energy and the edge energy for liver tumor segmentation. This model outperformed the Chan-Vese model and the geodesic level set model. In [31], Smeets et al. integrated fuzzy pixel classification with an edge-based LSM for liver tumor segmentation. The speed function for contour propagation was obtained from a nonlinearly filtered probabilistic distribution of liver tumor by supervised fuzzy clustering. This method surpassed the semiautomatic methods of other participants of MICCAI 2008 liver tumor segmentation challenge [32].

Existing LSMs can be categorized into two major classes: region-based models and edge-based models. However, both of them have inherent limitations for liver and liver tumor segmentation: (1) Considering liver segmentation, most used LSMs are edge-based models that introduce a speed function such as the balloon force term to shrink or expand the contour, since region-based models mostly are unable to detect the objective liver boundaries accurately. However, due to the existence of nonliver tissues such as vessels and tumors within the liver, edge-based LSMs with a small balloon force or insufficient iterations may not pass through the nonliver tissues, leading to undersegmentation. In contrast, if the balloon force is large or the iterations are excessive, the contours will pass through weak liver boundaries, leading to oversegmentation. So it is difficult to decide an appropriate group of the balloon force and evolution iterations for edge-based LSMs. (2) For liver tumor segmentation, the performance of edge-based LSMs relies on the precondition that liver tumors have clear and distinct edges. Unfortunately, liver tumors are often ambiguous, and edge-based LSMs will fail to identify the edges. On the other hand, although region-based LSMs are applicable to liver tumor segmentation, they have an inherent disadvantage that they are not competent to tackle complex liver tumors with low contrast and intensity inhomogeneities [33].

Rather than modifying or enhancing specific terms in the edge-based LSM or the region-based LSM to propose an improved LSM, we construct a unified level set framework by incorporating both edge information and region information of image to control the contour evolution. This framework is the core of the proposed methods for liver and liver tumor segmentation in this paper. Specifically, our proposed liver segmentation method mainly consists of three stages, a hybrid image preprocessing scheme to transform an original CT image into a binary image, region-growing to initially extract a rough liver region, and the unified LSM to refine the initial liver segmentation; our proposed liver tumor segmentation method mainly consists of three components, a region-based LSM proposed by Li et al. [33] coupled with hidden Markov random field and expectation-maximization (HMRF-EM) method [34, 35] to construct an enhanced edge indicator for the unified LSM, and the unified LSM to yield the final segmentation results.

Generally, LSMs can be applied to 2D segmentation by evolving curves, as well as 3D segmentation by evolving surfaces. The 3D level set method is theoretically a very nice solution, for it could produce a smoother boundary in the axial direction, leading to better incorporation of the 3D geometry. However, due to the complicated CT image conditions such as the noise and neighboring objects, the 3D segmentation is very sensitive to the initialization and suffers from the convergence to the local minima [36]. Therefore, the 3D level set often needs to combine an improved speed function incorporating prior knowledge such as shape prior and image features to receive acceptable results [37, 38]. In contrast, with a speed function that is not good enough, the 3D level set segmentation may get poorer results than the 2D level set segmentation, and the work of Street et al. [39] provides us with such an example. Besides, for liver segmentation in our study, users are allowed to terminate the level set evolution manually in some extreme cases (such as the case of almost complete absence of edge between the liver and adjacent tissues, where the LSM is prone to cause leakage due to lack of edge information) to ensure segmentation accuracy. And compared with the 3D surface evolution, users can observe the curve evolution more directly on the individual 2D slices, and so they can terminate the level set evolution more accurately, leading to more accurate results. Moreover, the higher dimension may bring more parameter settings, increase the computational burden, and reduce the segmentation efficiency [40]. Considering both accuracy and efficiency, we apply the 2D level set segmentation, which is more suitable for our study.

The anisotropy of abdominal CT data is mainly reflected in the various organs and tissues with complex anatomical structures and different intensities and the irregularly distributed noise within image. Our algorithms are able to resist and deal with the anisotropy of CT data, as well as the blurred edges, low contrast, and intensity inhomogeneities characterizing the CT data. In summary, the main contributions of this paper are as follows:

1. We propose a new unified LSM. This framework is more resistant to edge leakage than the single-information driven LSMs for liver segmentation and surpasses many other models for liver tumor segmentation. It is able to obtain more accurate results for liver and liver tumor segmentation.
2. We propose a hybrid image preprocessing scheme to convert the original CT image into a binary image. With this conversion, the median number of seed points required per CT image for region-growing to initially extract a rough liver region is 1 (range, 1 to 8). Besides, threshold setting and initial seed location setting become simpler, and there is no risk of oversegmentation.
3. To tackle more complex liver tumors, we construct an enhanced edge indicator for the unified LSM during liver tumor segmentation. With this development, our LSM can better handle the tumors with low contrast, ambiguous edges, and intensity inhomogeneities.
4. We extensively validate our methods with various datasets to show their accuracy and efficiency. The liver segmentation method outperforms other previous semiautomatic methods and requires less interaction on the SLIVER07 dataset. The liver tumor segmentation method is also competitive with other state-of-the-art methods on the 3Dircadb dataset.

The rest of this paper is organized as follows. A complete methodology of the proposed methods for liver and liver tumor segmentation is elaborated in Section 2. The performance of the proposed methods and method validation results are presented in Section 3. Finally, discussion and conclusion of the proposed methods are drawn in Section 4.

## 2. Methodology

**Figure 1:**
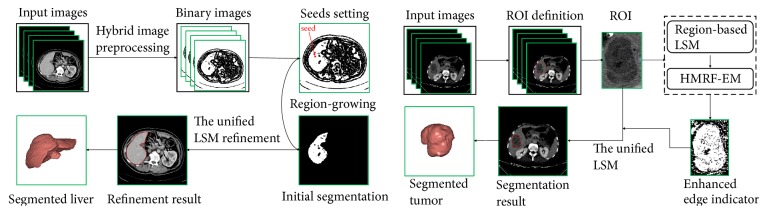
Flowchart of the proposed methods for liver and liver tumor segmentation. The left corresponds to liver segmentation, and the right corresponds to liver tumor segmentation.

An overview of the proposed methods for liver and liver tumor segmentation is illustrated in Figure 1. To be specific, we have the following:

1. For liver segmentation, a hybrid image preprocessing scheme consisting of an anisotropic filter [48], a scale-specific gradient magnitude filter [49], a nonlinear grayscale conversion, and a customized binary conversion is employed first to transform the input CT image into a binary image. Next, with manual setting of a few seed points on the obtained binary image, the following region-growing is performed to initially segment the liver. The unified LSM is proposed at last to refine the initial segmentation result.
2. For liver tumor segmentation, the user is required to define a tumor ROI manually first, where the following segmentation process will perform. Then the region-based LSM and the HMRF-EM algorithm are applied to construct an enhanced edge indicator for the unified LSM, which is performed at last to obtain the segmentation result.

### 2.1. Liver Segmentation

#### 2.1.1. Hybrid Image Preprocessing

**Figure 2:**
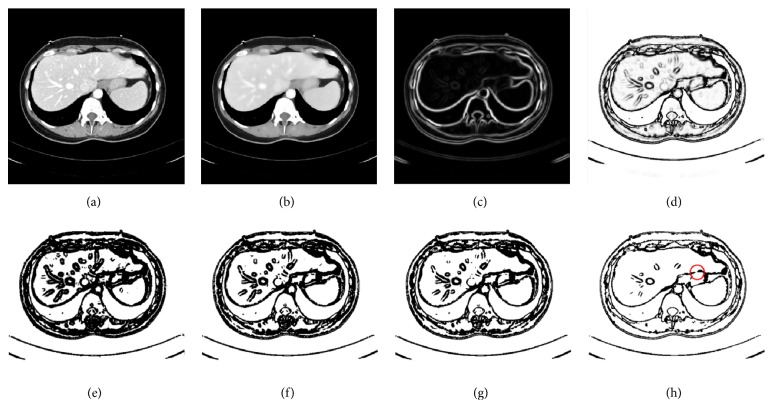
Results of hybrid image preprocessing: (a) original image; (b) after anisotropic filter; (c) after scale-specific gradient magnitude filter; (d) after nonlinear grayscale conversion; (e)-(h), respectively, indicate the results of binary conversion with *θ* = 1.1, *θ* = 1.2, *θ* = 1.7, and *θ* = 2.5 (part of the liver edge is eliminated within the red circle).

In the following, we give a detailed illustration of preprocessing. Instances of the results obtained by the hybrid image preprocessing are shown in Figure 2.

**Step 1 .**

Given an original image *f*, it is passed through a filter employing an anisotropic diffusion algorithm which is able to reduce the noise while preserving the boundary to obtain a noise-reduced CT image *f*_*I*_, as illustrated in Figure 2(b).

**Step 2 .**

The scale-specific gradient magnitude filter is employed on the noise-reduced CT image *f*_*I*_ to enhance the liver boundary. The scale of edge enhancement is controlled by the standard deviation *γ* of a Gaussian filter, expressed as

(1) $$\begin{array}{c} f_{E}\left(\;\gamma \;\right)=f_{I}*f_{G}=f_{I}*\frac{1}{{\left(2\pi \right)}^{1/2}\gamma }{exp}^{-\left(x^{2}+y^{2}\right)/2\gamma^{2}} \end{array}$$

where *∗* is a convolution operator. The standard deviation *γ* is stable; it was set to 0.5 [49], and its value remained constant in the experiment. Finally, calculate the magnitude of the image gradient at each pixel in image *f*_*E*_ by using the following differential operator to yield the gradient magnitude image *f*_*M*_, as shown in Figure 2(c):

(2) $$\begin{array}{c} f_{M}=\left|\;\nabla f_{E}\;\right|=\sqrt{{\left(\frac{\partial f_{E}}{\partial x}\right)}^{2}+{\left(\frac{\partial f_{E}}{\partial y}\right)}^{2}} \end{array}$$

**Step 3 .**

A nonlinear grayscale converter based on a sigmoid function is applied to enhance the contrast of the liver parenchyma, as illustrated in Figure 2(d). The sigmoid function is represented by

(3) $$\begin{array}{c} f_{s}=\frac{1}{1+exp^{\left(-\left(f_{M}-\beta \right)/\kappa \right)}} \end{array}$$

where *κ* is a parameter specifying the intensity range to be enhanced and *β* is a parameter controlling the center of the intensity range. They are stable, *κ* was set to -1.5, and *β* was set to 4 [49], and their values remained constant in the experiment.

**Step 4 .**

We propose a customized binarization method as the final preprocessing step to convert the image *f*_*s*_ into a binary image. The conversion function is defined by

(4) $$\begin{array}{c} f_{s}=\left\{\;f_{s}\geq \frac{\left(\max\left(f_{s}\right)+\min\left(f_{s}\right)\right)}{\theta }\;\right\} \end{array}$$

where max(*f*_*s*_) and min(*f*_*s*_) denote, respectively, the maximum and the minimum pixel intensity of *f*_*s*_, and *θ* > 1 is a parameter controlling the threshold.

The role of proposed binarization method is reflected in the following two aspects:

1. During Step 2, the scale-specific gradient magnitude filter not only enhances the liver boundaries, but also inevitably strengthens the extra noise within the liver. Binary conversion proposed here can reduce the noise enhancement. Results of noise reduction with binary conversion under different values of *θ* ranging from 1.1 to 2.5 are displayed in Figures 2(e)–2(h). It can be noted that, with the increase in values of *θ*, the degree of noise reduction increases. However, a large value of *θ* may eliminate part of the liver edge, as shown within the red circle in Figure 2(h). To find suitable *θ* values, a lot of tentative experiments were conducted. In the experiment, we found that *θ* was relatively stable; specifically, values of *θ* were almost the same for images of the same CT sequence; in contrast, for images from a different CT sequence, values of *θ* often required to be adjusted due to the differences among different CT sequences such as imaging conditions and pathological changes. We finally determined that the suitable range of *θ* values is 1.1 to 1.5.
2. In our study, conventional region-growing is used. Namely, a number of seed points are manually selected by users, and the initial region begins as the exact locations of these seed points. The regions are then grown from these seed points to adjacent pixel points depending on a homogeneity criterion. In our study, we define the homogeneity criterion by gray value. Specifically, a gray-level threshold *W* is introduced, if the absolute difference between the gray value of adjacent pixel point and the average gray value of the seed region is less than *W*, then the pixel point would be classified into the seed region; otherwise, skip and consider other pixel points. It is an iterated process until there are no pixel points satisfying the homogeneity criterion. It is known that conventional region-growing encounters the difficulties of threshold setting and initial seed location setting and that either of them with an inappropriate setting is prone to cause leakage, leading to oversegmentation. In contrast, the binarization method can tackle the mentioned problems since threshold setting and seed initial location setting become simple and reliable when region-growing is performed on binary images. Furthermore, fewer seed points would be required to extract a rough liver region; mostly, only one or two are needed per image.

**Figure 3:**
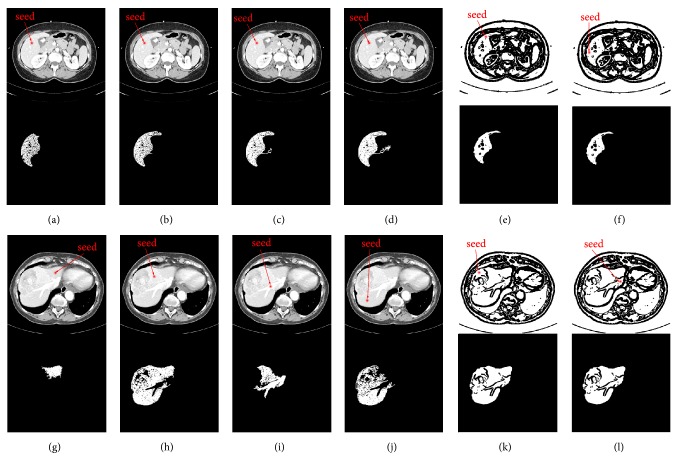
The comparison between region-growing for original CT image and region-growing for binary image. (a)-(b) indicate the original image M, (e)-(f) indicate the binary image of image M, (g)-(j) indicate the original CT image N, and (k)-(l) denote the binary image of image N. And images below (a)-(l) denote the corresponding results of region-growing. The initial seed points are denoted by red points, and *W* values of (a)-(d) were set to 0.04, 0.05, 0.06, and 0.07, respectively. *W* values of (g)-(j) were all set to 0.08, *W* values of (e) and (k) were both set to 0.3, and *W* values of (f) and (l) were both set to 0.8.

To clearly demonstrate the difference between region-growing for original CT image and region-growing for binary image, a comparison of them was made. We randomly selected two original CT images, defined them as image M and image N, and normalized their gray values to the range of 0 to 1. As illustrated in Figure 3, where Figures 3(a)–3(d) indicate the original CT image M, Figures 3(e) and 3(f) indicate the binary image of image M, Figures 3(g)–3(j) indicate the original CT image N, and Figures 3(k) and 3(l) denote the binary image of image N. And images below Figures 3(a)–3(l) denote the corresponding results of region-growing. To be specific, we did the following experiments: (1) We set the same initial seed locations and different gray-level threshold *W* values for the four images from Figures 3(a)–3(d); specifically, the initial seed points are denoted by red points, and *W* values of Figures 3(a)–3(d) were set to 0.04, 0.05, 0.06, and 0.07, respectively. From the results after region-growing, we can observe that when region-growing is applied to the original CT images, it is sensitive to the threshold. Improper thresholds do not yield accurate results, but we know it is difficult to set an appropriate threshold, which is often a tedious trial and error process. (2) We set the same gray-level threshold *W* values and different initial seed locations for the four images from Figures 3(g)–3(j); specifically, the initial seed points are denoted by red points, and *W* values of Figures 3(g)–3(j) were all set to 0.08. From the results after region-growing we can note that when region-growing is applied to the original CT images, it is sensitive to the initial seed location as well. Different initial seed locations may bring different results, but we know that the initial seed points are often set intuitively and randomly, which cannot guarantee the segmentation accuracy. (3) We set different initial seed locations and different gray-level threshold *W* values for Figures 3(e), 3(f), 3(k), and 3(l); specifically, the initial seed points are denoted by red points, the *W* values of Figures 3(e) and 3(k) were both set to 0.3, and the *W* values of Figures 3(f)–3(l) were both set to 0.8. From the results we can see that when region-growing is applied to the binary images, threshold setting and initial seed location setting become simple and reliable; namely, the initial seed points can be set anywhere in the white internally connected region, and any value in the range of 0 to 1 can be set as the gray-level threshold. Additionally, there is no risk of leakage.

#### 2.1.2. Initial Segmentation by Region-Growing

**Figure 4:**
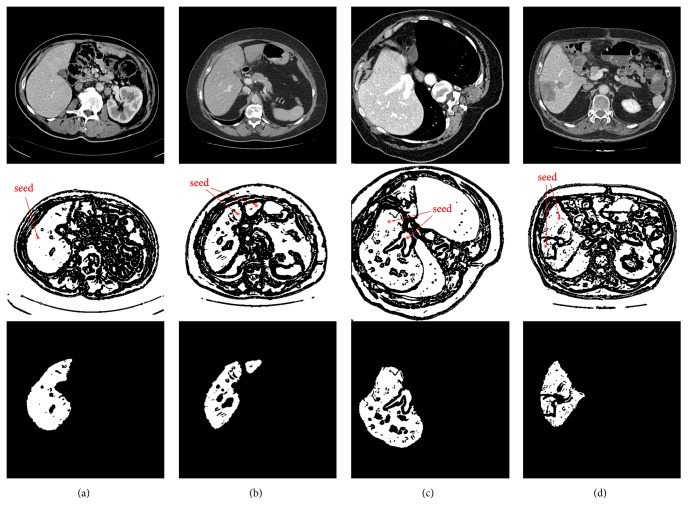
Four basic cases of seed settings and corresponding initial segmentation results: (a) a liver having an indiscrete region; (b) a liver having two discrete regions; (c) a liver having an inside vessel; (d) a liver having an inside liver tumor.

In this section, region-growing is performed on the obtained binary image to initially extract a rough liver region. The hybrid image preprocessing scheme provides a good condition for seed growth, as illustrated in Figure 2(g), where we can observe that, for an internally connected liver region in white, the entire liver region can be segmented via region-growing by setting one seed point at any location within the white area. However, in some cases, due to the presence of vessels and tumors within the liver, and the discretization of the liver, the whole liver region in binary image would be composed of several parts that are connected internally but not with others. So in order to extract the whole liver region as completely as possible, users should set a seed point within each of those parts as much as possible for region-growing. For the sake of representativeness, four basic examples of seed point settings and corresponding derived results are displayed in Figure 4, where column (a) denotes a liver having an indiscrete region, and one seed point is required for region-growing. In column (b), there is a liver having two discrete regions, and two seed points are required. Column (c) indicates that two seed points are needed for a liver having an inside vessel while two seed points are required as well for a liver having an inside tumor in column (d).

Note that region-growing for a liver in a more complex case (such as a liver having discrete regions and inside tumors) may require more seed points to ensure that all the liver areas could be segmented. According to statistics, in our liver segmentation experiment, the median number of seed points required per CT image for region-growing to initially extract a rough liver region is 1 (range, 1 to 8), and more details can be found in Section 3.5.2.

#### 2.1.3. Liver Segmentation Refinement

From Figure 4 we can notice that the outcomes of region-growing do not meet our expectations of the final segmentation results, and they need to be further refined. LSMs are good choices to smooth and refine the initial segmentation [50].

The region-based LSM is not preferred for liver segmentation as it is often unable to detect the objective liver boundaries correctly. And the edge-based LSM encounters the difficulty of setting an appropriate group of the balloon force and iterations. In order to tackle these limitations, we propose a unified double-information driven LSM for liver segmentation. With an enhanced edge indicator, this level set framework is applicable for complex liver tumor segmentation as well. Compared with other two single-information driven LSMs, our LSM has proved to be able to adapt to a larger balloon force and more iterations (the comparisons can be found in Section 3.3.2). With such superiority, we can set a large balloon force and enough iterations for the unified LSM. In this way, our LSM not only can ensure the contour evolves to the objective liver edges eventually, but also is resistant to boundary leakage to obtain more accurate segmentation. In the following, we first introduce the unified LSM and its implementation for liver segmentation refinement.

*(A) The Unified LSM*. The general formulation of the unified LSM follows the outline of the original distance regularized level set evolution (DRLSE) model [51].

Let *Ω* ⊂ *R*^2^ be the image domain and *I*: *Ω* → *R* be a given image. Within the level set formulation, the evolving contour in the image plane, denoted by *C*, is represented by the zero level set *C*(*t*) = {(*x*, *y*)∣*φ*(*x*, *y*, *t*) = 0} of a level set function (LSF) *φ*(*x*, *y*, *t*).

We define an energy functional *ε*(*φ*) by

(5) $$\begin{array}{c} \varepsilon \left(\;\varphi \;\right)=\mu D\left(\;\varphi \;\right)+\varepsilon_{ext}\left(\;\varphi \;\right) \end{array}$$

where *ε*_ext_(*φ*) is the external energy. *μ* > 0 is a coefficient of the level set regularization term *D*(*φ*), which eliminates the reinitialization of the LSM and is defined by

(6) Dφ=∫Ωp∇φdxdy

(7) p∇φ=12π21−cos⁡2π∇φ,∇φ≤11 2∇φ−12,∇φ≥1

where *p*(|∇*φ*|) is a double-well potential function for distance regularization, whose advantage is that a binary step function can be used as an initial LSF.

We use an original edge indicator function *g* as the edge-based image information to define the external energy.

(8) $$\begin{array}{c} g=\frac{1}{1+{\left|\nabla G_{\sigma }*I\right|}^{2}} \end{array}$$

where *G*_*σ*_ is a Gaussian kernel with a standard deviation *σ*. ∇*G*_*σ*_*∗I* is a convolution to reduce the noise of *I*. The value of *g* is large in the homogeneous region to speed up the contour propagation and becomes small around the distinct boundary to slow down the contour evolution.

Then *ε*_ext_(*φ*) can be defined by

(9) εextφ=λLφ+αAφ=λ∫Ωgδφ∇φdxdy+α∫ΩgH−φdxdy

where *λ* and *α* are constant coefficients and *δ* and *H* are the Dirac delta function and the Heaviside function, respectively. *L*(*φ*) computes the line integral and *A*(*φ*) controls the curve evolution.

Zhang et al. [52] reconstructed a novel region-based balloon force called Signed Pressure Force (SPF), represented by

(10) $$\begin{array}{c} SPF\left(\;I\left(\;x,y\;\right)\;\right)=\frac{I\left(x,y\right)-\left(c_{1}+c_{2}\right)/2}{\max\left(\left|I\left(x,y\right)-\left(c_{1}+c_{2}\right)/2\right|\right)} \end{array}$$

where *c*_1_ and *c*_2_ are two constants that are average intensities inside and outside the contour, respectively, and can be derived by C-V model [53].

For the given image *I* on the domain *Ω*, the C-V model is formulated by minimizing the following energy function:

(11) E=λ1∫insidecIx,y−c12dxdy+λ2∫outsidecIx,y−c22dxdy,x,y∈Ω

where *λ*_1_ and *λ*_2_ are constant coefficients.

With the LSM, we assume

(12) c=x,y∈Ω:φx,y=0insidec=x,y∈Ω:φx,y>0outsidec=x,y∈Ω:φx,y<0

By minimizing *E* in (11), we get *c*_1_ and *c*_2_ as follows:

(13) c1φ=∫ΩIx,y·Hφdxdy∫ΩHφdxdy

(14) c2φ=∫ΩIx,y·1−Hφdxdy∫Ω1−Hφdxdy

where *H* is the Heaviside function.

We integrate the SPF to *ε*_ext_(*φ*) to get a new external function, represented by

(15) εunifiedφ=λ∫Ωgδφ∇φdxdy+α∫Ωg·SPFIx,yH−φdxdy

where *α* is a parameter controlling the magnitude of balloon force.

Thus, the unified LSM could be expressed as

(16) $$\begin{array}{c} \varepsilon \left(\;\varphi \;\right)=\mu D\left(\;\varphi \;\right)+\varepsilon_{unified}\left(\;\varphi \;\right) \end{array}$$

*(B) The Unified LSM Refinement*. Let the binary image obtained from region-growing be *I*_0_. Thanks to the double-well function *p*(|∇*φ*|) in DRLSE frame, a binary step function is allowed to initialize the LSF *G*_0_ directly. The initialization function is defined by

(17) $$\begin{array}{c} G_{0}=\omega \cdot \left(\;I_{0}-\frac{1}{2}\;\right) \end{array}$$

where *ω* is a parameter controlling the width of Signed Distance Function (SDF). As suggested in [51], *ω* should be chosen from the range *ω* ≥ 2. We set *ω* = 4 in our study.

As for the stop criterion of the LSM refinement for liver segmentation, on the one hand, the contour propagation will stop if the maximum number of iterations is reached. On the other hand, the user is allowed to terminate the LSM evolution manually. This additional manual termination is proposed to ensure the unified LSM could bring expected segmentation even in extreme cases, such as the case of almost complete absence of edge between the liver and adjacent tissues, where the LSM is prone to cause leakage due to lack of edge information. Note that this additional operation has no significant effect on the interaction burden since the proportion of extreme cases is small (a detailed interaction analysis can be found in Section 3.5).

**Figure 5:**
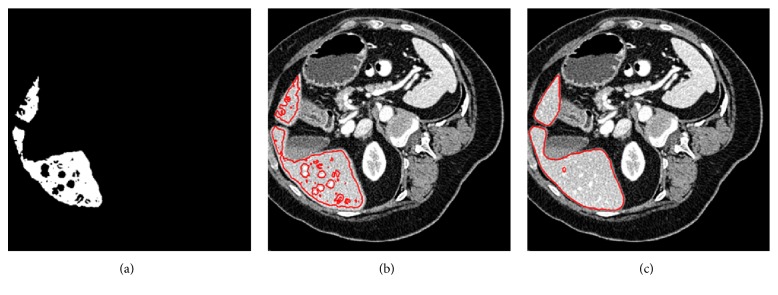
Examples of the unified LSM initialization and refinement: (a) binary image obtained from region-growing; (b) LSM initialization; (c) the result of refinement.

Examples of the unified LSM initialization and refinement are illustrated in Figure 5, where Figure 5(a) shows the binary result obtained by region-growing, Figure 5(b) shows the LSM initialization, and Figure 5(c) shows the refinement result. It can be seen that the unified LSM could bring an expected segmentation result. Moreover, due to the noise within the liver, there would be holes inside the segmentation results in some cases, such as the case shown in Figure 5(c). We employ a morphological filling scheme to deal with this problem.

### 2.2. Liver Tumor Segmentation

#### 2.2.1. ROI Definition

**Figure 6:**
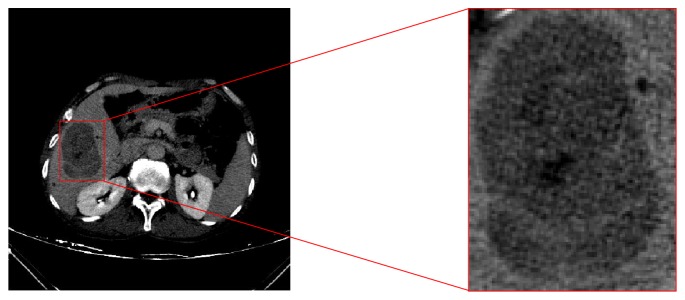
An example of a ROI definition.

The purpose of a ROI definition is to limit the tumor segmentation to the selected region. Advantages of the ROI definition are as follows: (1) locations of liver tumors are not determined, and a ROI provides location information; (2) a ROI allows to only consider liver and tumor tissues and avoid other tissues in the abdomen; (3) a ROI reduces the number of pixels and improves the efficiency of segmentation. In our study, we use a rectangle to define a ROI. In addition, it is important to notice that if we consider the case of tumors located on the liver borders and adjacent to other structures in the abdomen, then the choice of a ROI exceeding the limits of the tumor may affect the segmentation result [54]. Thus, a ROI must be selected as close as possible to the tumor to avoid such problems. An example of a ROI definition is shown in Figure 6.

#### 2.2.2. Construction of the Enhanced Edge Indicator

Original edge indicator *g* in (8) can be applied properly as the edge information for the unified LSM to segment the liver since livers mostly have more clear boundaries than liver tumors. So in order to improve the performance of the unified LSM for liver tumor segmentation, instead of using the original *g*, we construct an enhanced edge indicator based on a combination of the region-based LSM proposed by Li et al. [33] and the HMRF-EM scheme. The main advantage of this region-based LSM is that it can handle intensity inhomogeneities well. Additionally, the HMRF-EM algorithm is known to be competent to deal with low-contrast images. Therefore, the unified LSM can be endowed with the mentioned abilities to tackle more complex liver tumors. The optimized edge indicator can be obtained through the following three parts.

*(A) The Region-Based LSM*. Let *Ω* ⊂ *R*^2^ be the image domain and *I*: *Ω* → *R* be a given image. In our study, we make an assumption of two-phase segmentation. Namely, the image domain is segmented into two disjoint regions *Ω*_1_ (liver tissue) and *Ω*_2_ (liver tumor). The two-phase LSM is formed by the following steps.

**Step 1 .**

Assume the observed image *I* can be modeled as

(18) $$\begin{array}{c} I=b\cdot J+n \end{array}$$

where *J* is the true image, *b* is the bias field, which is the component resulting in the intensity inhomogeneities, and *n* is extra noise. This is a general model of images with intensity inhomogeneities.

**Step 2 .**

Derive a local intensity clustering property. Let **y** denote each point of *Ω*, and consider each point **y** ∈ *Ω* as a center with a radius *ρ* to form a circular neighborhood, which is defined by *O*_**y**_ = {**x** : |**x** − **y**| ≤ *ρ*}. The partition of *Ω*_1_ and *Ω*_2_, respectively, induces a partition of the neighborhood *O*_**y**_; i.e., {*O*_**y**_∩*Ω*_*i*_}_*i*=1_^2^ forms a partition of *O*_**y**_. Because the bias field *b* is assumed to be slowly varying, and according to the image model in (18), we have

(19) $$\begin{array}{c} I\left(\;x\;\right)\approx b\left(\;y\;\right)c_{i}+n\left(\;x\;\right),\;x\in O_{y}\cap \Omega_{i},\;\;i=1,2 \end{array}$$

where *c*_*i*_ is constants in disjoint region *Ω*_*i*_ and *n*(**x**) is additive zero-mean Gaussian noise. Therefore, the intensities in the set

(20) $$\begin{array}{c} I_{y}^{i}=\left\{\;I\left(\;x\;\right):x\in O_{y}\cap \Omega_{i},\;\;i=1,2\;\right\} \end{array}$$

form a cluster with cluster center *m*_*i*_ ≈ *b*(**y**)*c*_*i*_, *i* = 1,2 which could be regarded as samples drawn from a Gaussian distribution with mean *m*_*i*_.

**Step 3 .**

Apply the standard K-means clustering to classify the local intensities and derive a level set energy formulation. The domain of the image is segmented into two disjoint regions *Ω*_1_ and *Ω*_2_, and we use a LSF *ϕ* to represent the two regions; specially, its signs can define the two disjoint regions: *Ω*_1_ = {**x** ∈ *Ω* : *ϕ*(**x**) > 0}, and *Ω*_2_ = {**x** ∈ *Ω* : *ϕ*(**x**) < 0}. And the two regions can be represented with their membership functions defined by *M*_1_(*ϕ*) = *H*(*ϕ*) and *M*_2_(*ϕ*) = 1 − *H*(*ϕ*), respectively, where *H*(*ϕ*) is the Heaviside function. For *ξ* = 2, the level set energy formulation can be defined by

(21) ε=∫∑i=1ξ∫ΩiKy−xIx−byci2dy·Miϕxdx

where *K* is a kernel function, which is defined by a truncated Gaussian function:

(22) $$\begin{array}{c} K\left(\;u\;\right)=\left\{\begin{array}{cc} \frac{1}{a}e^{-{\left|u\right|}^{2}/2\tau^{2}}, & \text{for }\left|u\right|\leq \rho \\ 0, & otherwise \end{array}\right. \end{array}$$

where *a* is a normalization constant, *τ* is the standard deviation of the Gaussian function, and *ρ* is the radius of the neighborhood *O*_**y**_. We set *a* = 1, *τ* = 4, and *ρ* = 3 in our study.

**Step 4 .**

By using the DRLSE frame and adding the energy *ε* in (21) into the frame, we get the final level set formulation that is expressed as

(23) Fϕ,c,b=εϕ,c,b+v∫Ω∇Hϕdx+δ∫Ωq∇ϕdx

where the third term and the last term are the regularization terms and the potential function *q* is defined by

(24) $$\begin{array}{c} q\left(\;\nabla \phi \;\right)=\frac{1}{2}{\left(\;\nabla \phi -1\;\right)}^{2} \end{array}$$

Due to the robustness of this LSM to contour initialization, we use a random initialization mechanism to initialize the contour automatically, avoiding manual labor [33].

**Figure 7:**
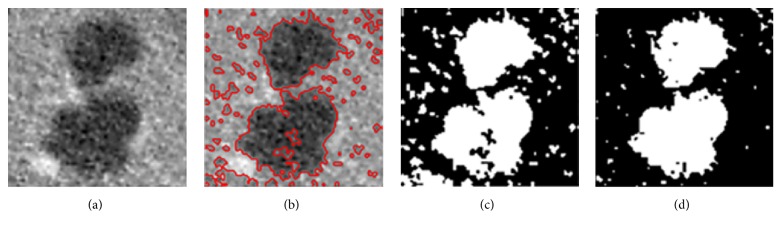
Results of the region-based LSM and the HMRF-EM scheme: (a) an original ROI; (b) the region-based LSM segmentation; (c) the binary mask of (b); (d) the improved binary classification obtained via the HMRF-EM algorithm.

An example of a segmentation result obtained through this LSM is shown in Figure 7, where Figure 7(a) shows an original ROI, Figure 7(b) shows the segmentation result denoted by red lines, and Figure 7(c) shows the binary mask of Figure 7(b). The binary mask would be used as the initial state condition for the following HMRF-EM process.

*(B) The HMRF-EM Algorithm*. The HMRF-EM algorithm is a stochastic process. Considering that the underlying stochastic process of a hidden Markov model (HMM) is a Markov random field (MRF) instead of a Markov chain, this special model is referred to as a HMRF model. The EM algorithm is employed to solve maximum likelihood (ML) estimation of the parameters. By incorporating both the HMRF model and the EM algorithm, a mathematically sound HMRF-EM framework is obtained. Note that the EM algorithm is a local minimization method, which can be trapped in local minimum. So with an initial condition far from normal, the EM procedure is likely to give wrong segmentation [35]. To avoid this, we use the binary mask obtained from the last LSM as the initial condition. Besides, initialization like this is able to connect the two independent methods to combine their advantages. The HMRF-EM framework employed in our study is discussed below.

Given an image *I*, assume a set *β* = (*β*_1_, *β*_2_,…, *β*_*N*_), where each *β*_*i*_ is the intensity of a pixel in *I*, and *N* is the total number of pixels. In our HMRF-EM process, the target is to segment the image into two parts. To be specific, a binary segmentation is required. Let *L* = {0,1} be the set of two labels, and each pixel in *I* is assigned to label 0 or 1. Among the pixels, those with the same labels would form a configuration of labels *α* = (*α*_1_, *α*_2_,…, *α*_*N*_). We seek the labeling *α*^*∗*^ as an estimation of the true labeling *α*^*T*^ according to the maximum a posteriori (MAP) principle, expressed as

(25) $$\begin{array}{c} \alpha^{*}=\underset{\alpha }{\mathrm{argmax}}\left\{\;P\left(\;\beta \;|\;\alpha ,\theta \;\right)P\left(\;\alpha \;\right)\;\right\} \end{array}$$

where *P*(*α*) is the prior probability equivalent to Gibbs distribution and could be written as

(26) $$\begin{array}{c} P\left(\;\alpha \;\right)=\frac{1}{Z}\exp\left(\;-U\left(\;\alpha \;\right)\;\right) \end{array}$$

where *Z* is a normalization factor and *U*(*α*) is the prior energy function.

And *P*(*β*∣*α*, *θ*) in (25) is the joint likelihood probability given by

(27) $$\begin{array}{c} P\left(\;\beta \;|\;\alpha ,\theta \;\right)=\underset{i}{\prod }P\left(\;\beta_{i}\;|\;\alpha ,\theta \;\right)=\underset{i}{\prod }P\left(\;\beta_{i}\;|\;\alpha_{i},\theta_{\alpha_{i}}\;\right) \end{array}$$

where *P*(*β*_*i*_∣*α*_*i*_, *θ*_*α*_*i*__)is a Gaussian distribution with a parameter *θ*_*α*_*i*__ = (*μ*_*α*_*i*__, *σ*_*α*_*i*__^2^).

Assume *θ* = {*θ*_*l*_∣*l* ∈ *L*} is unknown, and it could be obtained via the iterative EM algorithm. In the EM algorithm, *α*^*∗*^ could be solved by minimizing the total posterior energy according to the MAP estimation:

(28) α∗=argmaxα⁡Pβ ∣ α,θPα=argminα⁡Uβ ∣ α,θ+Uα

where *U*(*α*) and *U*(*β*∣*α*, *θ*), respectively, are defined by

(29) Uα=∑c∈CVcα,Uβ ∣ α,θ=∑iβi−μαi22σαi2+ln⁡σαi

where *V*_*c*_(*α*) is the clique potential and *C* is the set of all possible cliques.

With these assumptions and conditions mentioned above, the HMRF-EM algorithm used in this paper could be described as follows, and the binary mask provides the initial labels *α*^(0)^ for the MAP estimation and the initial parameters *θ*^(0)^ for the EM algorithm [34]:

(1) Start with the initial condition provided by the binary mask.

(2) Calculate the likelihood distribution *P*^(*t*)^(*β*_*i*_∣*α*_*i*_, *θ*_*α*_*i*__).

(3) Estimate the class labels with current *θ*^(*t*)^ via MAP estimation:

(30) αt=argmaxα⁡Pβ ∣ α,θPα=argminα⁡Uβ ∣ α,θt+Uα

(4) Calculate the posterior distribution for all pixel intensities *β* and all *l* ∈ *L*:

(31) $$\begin{array}{c} P^{\left(t\right)}\left(\;l\;|\;\beta_{i}\;\right)=\frac{\left(g^{\left(t\right)}\left(\beta_{i};\theta_{l}\right)\cdot P^{\left(t\right)}\left(l\;|\;{\alpha_{N_{i}}}^{\left(t\right)}\right)\right)}{P^{\left(t\right)}\left(\beta_{i}\right)} \end{array}$$

where $g\left(\beta_{i};\theta_{l}\right)=\left(1/\sqrt{2\pi {\sigma_{l}}^{2}}\right)\exp\left(-{\left(\beta_{i}-\mu_{l}\right)}^{2}/2{\sigma_{l}}^{2}\right)$ is a Gaussian distribution function.

(5) Update parameters with *P*^(*t*)^(*l*∣*β*_*i*_):

(32) μlt+1=∑iPtl ∣ βiβi∑iPtl ∣ βiσlt+12=∑iPtl ∣ βiβi−μlt+12∑iPtl ∣ βi

(6) *t* ← *t* + 1 and repeat from (2) until enough iterations are performed.

Through the HMRF-EM framework, an improved binary classification having advantages of the two mentioned methods is obtained, as illustrated in Figure 7(d). It can be observed that the binary classification well indicates the edge position.

*(C) The Enhanced Edge Indicator*. Although the binary result of the HMRF-EM algorithm can well indicate the edge position, it is not fit to be the edge indicator because a drawback of it is that evolution of the level set would completely stop at the binary edge due to the gray values of black area being 0, making the segmentation unsmooth and inaccurate. Thus, inspired by the work of Li et al. [50], we obtain the enhanced edge indicator through a maximum operation, which is defined by

(33) $$\begin{array}{c} g_{enhanced}=\max\left(\;g_{binary},g\;\right) \end{array}$$

where *g*_enhanced_ is the enhanced indicator, *g*_binary_ is the binary result, and *g* is the original indicator.

**Figure 8:**
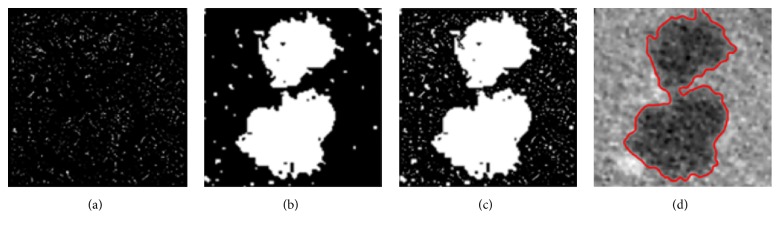
(a) original *g*; (b) *g*_binary_; (c) *g*_enhanced_; (d) the segmentation result.

Figure 8(a) shows the original *g*, Figure 8(b) shows *g*_binary_, and Figure 8(c) shows *g*_enhanced_. It can be noted that the original *g* is vague that can hardly provide us with clear edge information, but an advantage of it is that its gray values of black area are close to 0 instead of being 0, which ensures that the level set always has a speed, whereas *g*_binary_ is the opposite. So neither of them is fit to be the edge indicator. Fortunately, the maximum operation is able to endow *g*_enhanced_ with the advantages of the first two. That is to say, *g*_enhanced_ not only provides clear edge information, allowing rapid evolution of the level set inside the tumor, but also prevents the evolution from terminating completely at the edge to make the result smoother and more accurate, as shown in Figure 8(d).

#### 2.2.3. The Unified LSM Segmentation

By replacing the original *g* with the enhanced *g*_enhanced_ in (15), the unified LSM for liver tumor segmentation is represented by

(34) εφ=μDφ+λ∫Ωgenhancedδφ∇φdxdy+ϑ∫Ωgenhanced·SPFIx,yH−φdxdy

where *ϑ* is a parameter controlling the magnitude of the balloon force, the same as *α* in (15).

The unified LSM for liver tumor segmentation requires manual initialization, and the initial contour is preferred to be located inside the liver tumor since the enhanced edge indicator allows faster contour propagation in white area. In our study, we initialize the LSM with a rectangle inside the liver tumor. Additionally, unlike the liver segmentation, no additional interaction is required to terminate the level set evolution due to the enhanced edge indicator, whereas contour propagation will stop when the maximum number of iterations is reached.

**Figure 9:**
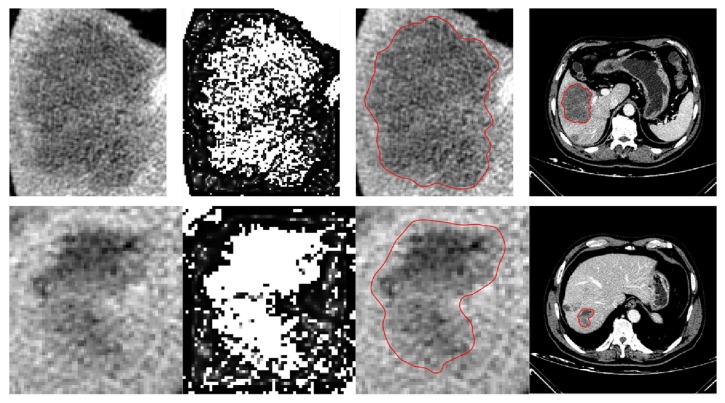
Two examples of challenging liver tumor segmentation: (rows) from top to bottom: a tumor case with an ambiguous and variable edge and a tumor case with low contrast and intensity inhomogeneities; (columns) from left to right: the original ROIs, the enhanced edge indicators, the segmentation results denoted by red lines, and the results shown in full images.

Successful segmentation examples of two challenging liver tumors are displayed in Figure 9, where the first row indicates a tumor case with an ambiguous and variable edge, and the second row indicates a more complex tumor case with low contrast and intensity inhomogeneities. And columns from left to right indicate, respectively, the original ROIs, the enhanced indicators, the segmentation results denoted by red lines, and the results shown in full images.

## 3. Evaluation and Results

### 3.1. Datasets

The proposed methods were implemented in C++/mex and Matlab environment on a Windows-based computer with an i5-2400 3.1GHz CPU, AMD Radeon HD 6450 GPU, and 6GB RAM. Details of the used validation data are given below:

1. For liver segmentation, the applied data came from the SLIVER07-Train database and the 3Dircadb database. The SLIVER07-Train database contains 20 contrast-enhanced CT volumes with standard segmentation. All the volumes have an in-plane resolution of 512 × 512 pixels. The inner-slice pixel spacing varies from 0.57 to 0.82 mm, and the slice thickness varies from 0.7 to 5.0 mm. The 3Dircadb database contains 20 CT volumes and corresponding ground truth as well; the pixel spacing and slice thickness vary from 0.56 to 0.87 mm and 1.25 to 4 mm, respectively, with the in-plane resolution of 512 × 512 pixels in all cases.
2. Three datasets were used for liver tumor segmentation method validation. They are a local dataset acquired at Ningbo Li Hui-Li hospital, China, the MIDAS dataset provided by the Imaging Science and Information Systems (ISIS), and the 3Dircadb dataset. There are 10 liver tumors in the hospital data coming from 3 patients; each image has a matrix size of 512 × 512 pixels, with an in-plane pixel size of 0.68 to 0.69 mm and the slice thickness of 2 mm. And the hospital data was used first for method training to find optimal parameters in our study. The MIDAS data provides 4 liver tumors coming from 4 patients. The matric size varies from 177 × 177 to 189 × 189 pixels. And the in-plane pixel size and slice thickness are 1.73 to 1.85 mm and 1.73 to 1.85 mm, respectively. The 3Dircadb data contains 121 liver tumors. Each slice has a matric size of 512 × 512 pixels, with an in-plane pixel size of 0.56 to 0.87 mm and the slice thickness of 1.25 to 4 mm.

### 3.2. Evaluation Measures

For accuracy evaluation, five evaluation measures were used to compare each segmentation result with its corresponding reference segmentation. They are volumetric overlap error (VOE, %), relative absolute volume difference (RVD, %), average symmetric surface distance (ASD, mm), root mean square symmetric surface distance (RMSD, mm), and maximum symmetric surface distance (MSD, mm) [2]. Calculating different measures for average and maximum errors will convey more segmentation information than just using one measure. All measures are larger than or equal to zero; a value of 0 corresponds to an exact match with the reference segmentation, which means a larger value corresponds to a poorer segmentation result.

### 3.3. Liver Segmentation

#### 3.3.1. Method Training and Parameter Setting

Here, we give the values of the most important parameters. We set *λ*_1_ = *λ*_2_ = 1 in (11) [53], *λ* = 5 in (15), and *μ* = 0.04 in (16) [51]. To determine an optimal group of the balloon force and iterations *t* for the unified LSM, we randomly selected 10 CT volumes from the SLIVER07-Train database for method training. Firstly, with a tentative experiment conducted, we decided 9 groups of *α* and *t* as candidates for the optimal group. Then with the same initialization, 9 unified LSMs with different settings were performed, respectively, on the 10 volumes to yield corresponding segmentation results. The segmentation results were compared with the ground truth using RVD, and a smaller absolute value of RVD meant a better setting. Each group and its corresponding RVD are listed in Table 1, where we can note that *α* = 5, *t* = 150 and *α* = 10, *t* = 100 are the best and second best groups, whose RVD are 0.85% and 1.05%, respectively. We chose *α* = 10, *t* = 100 as the optimal group in our study because it required fewer iterations while its RVD value was close to that of the former.

#### 3.3.2. Level Set Comparison

In this section, to demonstrate the superiority of the proposed unified LSM for liver segmentation, we made three comparisons of the unified LSM and other two single-information driven LSMs. The three LSMs are all in the DRLSE framework, respectively, represented by

(1)

(35) εunified-DRLSEφ=μDφ+λ∫Ωgδφ∇φdxdy+α∫Ωg·SPFIx,yH−φdxdy

(2)

(36) εedge-DRLSEφ=μDφ+λ∫Ωgδφ∇φdxdy+α∫Ωg·H−φdxdy

(3)

(37) εregion-DRLSEφ=μDφ+λ∫Ωgδφ∇φdxdy+α∫ΩSPFIx,yH−φdxdy

where the unified-DRLSE is the proposed unified LSM, the edge-DRLSE is edge-based, which is driven by the original edge indicator *g*, and the region-DRLSE is region-based, which is driven by the SPF.

**Figure 10:**
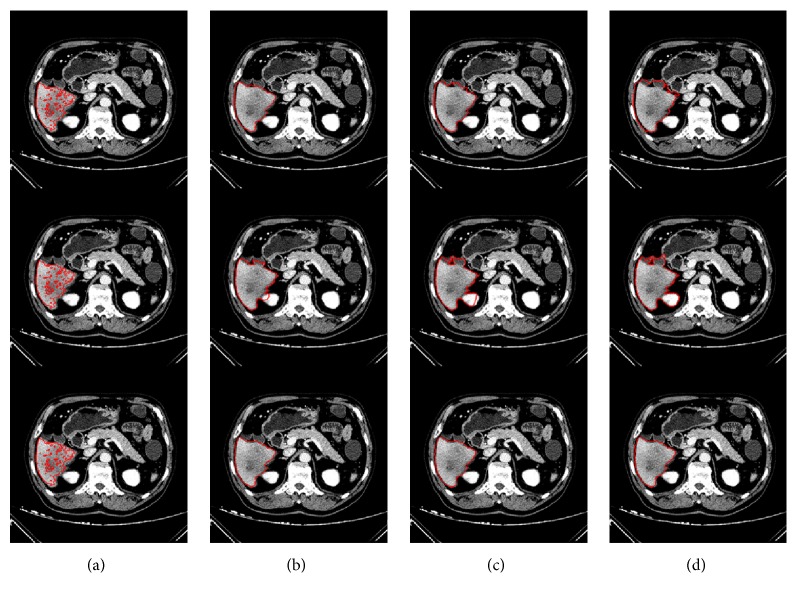
First comparison: (rows) from top to bottom: the results of the edge-DRLSE, the region-DRLSE, and the unified-DRLSE, respectively; (columns): (a) level set initialization; (b) results under iteration 50; (c) results under iteration 100; (d) results under iteration 150.

*(a) First Comparison*. To make the comparison result more noticeable, we set larger *α* and more iterations to all three LSMs. Specifically, we set *α* = 20 to both the edge-DRLSE and the region-DRLSE and set *α* = 30 to the unified-DRLSE. The iterations *t* of three LSMs were all set to 150. With the same initialization, three LSMs were performed on a randomly selected CT image. Segmentation results are illustrated in Figure 10, where three rows from top to bottom indicate the results of, respectively, the edge-DRLSE, the region-DRLSE, and the unified-DRLSE. And column (a) indicates the level set initialization, while columns (b) to (d) denote the segmentation results under iteration 50, iteration 100, and iteration 150, respectively.

From the results, we can note that, for the edge-DRLSE, there is already an obvious leakage when the number of iterations reaches 50. As the number of iterations increases, the leakage becomes more and more serious. The region-DRLSE is unable to detect the liver boundary correctly, leading to global segmentation. In contrast, as for the unified-DRLSE that is under a larger balloon force, we can observe that its leakage changes imperceptibly with the increase in iterations. This qualitative comparison shows the unified-DRLSE is able to adapt to a larger balloon force and more iterations.

*(b) Second Comparison*. For this comparison, we randomly selected other two CT images and defined them as image A and image B, respectively. To make the comparison result more noticeable as well, we set *α* = 10, *t* = 200 and *α* = 20, *t* = 200, respectively, to all three LSMs. With the same initialization, three LSMs with two different settings were performed, respectively, on image A and image B. We obtained the segmentation results of three LSMs under every iteration and compared them with the ground truth via VOE and RVD.

**Figure 11:**
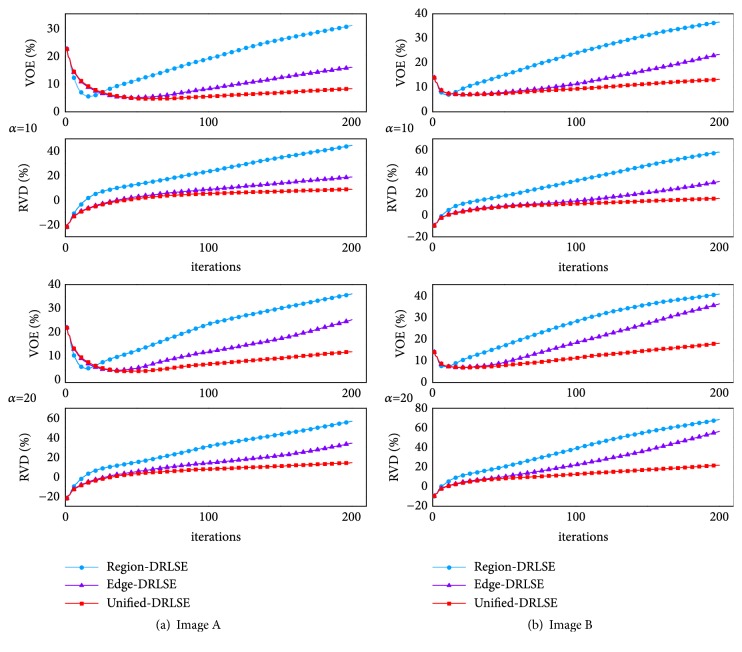
Second comparison: we set *α* = 10, *t* = 200, and *α* = 20, *t* = 200, respectively, to three LSMs. Segmentation results obtained under every iteration were compared with the ground truth via VOE and RVD.

As illustrated in Figure 11, where we can see that, with the increase in iterations, the curves of VOE and RVD belonging to the unified-DRLSE are always under those of other two single-information driven LSMs and are more flat as well. As aforementioned, smaller values of VOE and RVD correspond to a better segmentation result. So this quantitative comparison proves that the unified-DRLSE not only is resistant to a larger balloon force and more iterations, but also is able to obtain more accurate segmentation results.

**Figure 12:**
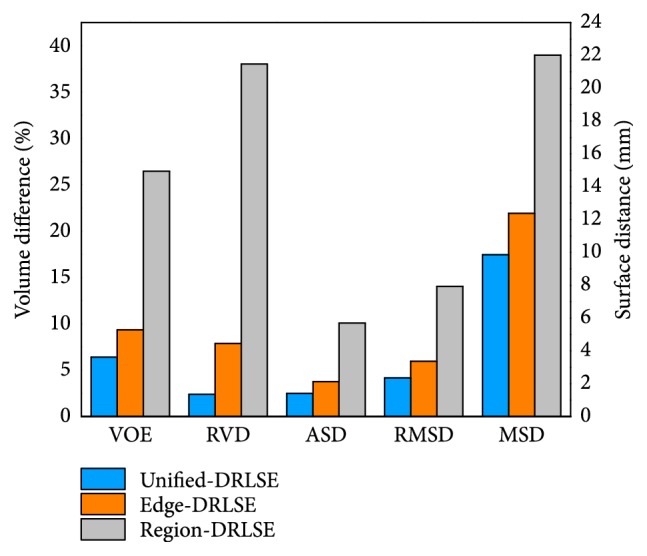
Third comparison: bar chart: three LSMs set with *α* = 15 and *t* = 100 were performed on all 40 CT volumes. Segmentation results were compared with the ground truth via five measures.

*(c) Third Comparison*. To make the comparison result have more statistical significance, three LSMs were performed, respectively, on all 40 CT volumes in this comparison. All LSMs were set with *α* = 15 and *t* = 100. The segmentation results were compared with the ground truth via all five measures. As illustrated in Figure 12, where the unified-DRLSE gets the best segmentation, whose measure values are 6.43%, 2.40%, 1.41 mm, 2.36 mm, and 9.85 mm. The edge-DRLSE yields the second best segmentation, whose measure values are 9.35%, 7.90%, 2.13 mm, 3.36 mm, and 12.38 mm while the region-DRLSE gets the worst values of 26.46%, 38.04%, 5.70 mm, 7.92 mm, and 22.01 mm.

Note that, in order to remain objective during the three comparisons, no additional manual termination was added; instead, contour evolution stopped when the maximum number of iterations was reached. Results of the above three comparisons were in line with our assumptions. That is, the region-DRLSE often failed to observe the objective liver edges accurately, leading to the worst performance among the three. The edge-DRLSE was able to detect the liver boundaries, but it was unable to stay stable under a larger balloon force or more iterations. So it outperformed the region-DRLSE but was not as good as the unified-DRLSE. The unified LSM achieved the best performance, which proves that combining more image information is able to make the model more stable and lead to more accurate segmentation.

#### 3.3.3. Validation Results

The proposed liver segmentation method was validated with the two public datasets via five measures. Validation results are illustrated in Table 2.

**Figure 13:**
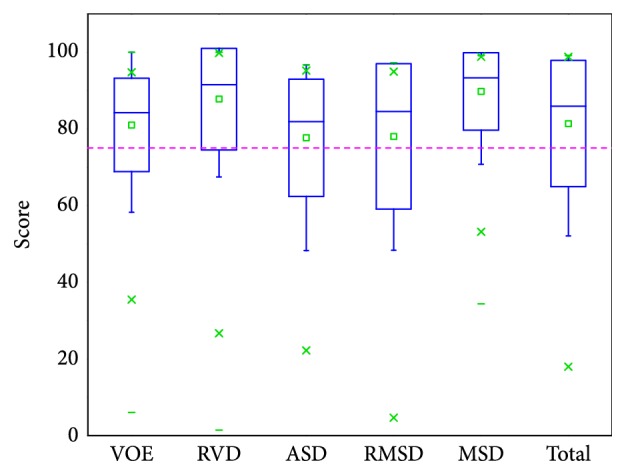
Scores of the SLIVER07 dataset, presented as a boxplot, where squares indicate the mean values. Score of average interobserver variability (75) is shown with dashed line for reference.

For the SLIVER07 challenge data, independent delineations were provided by the organization of the challenge. Based on the comparison a score was given to each validation measure and to each segmentation result of the challenge data. A score of 100 was assigned to the perfect segmentation, i.e., a value of 0 for each validation measure. Reference values of VOE = 6.4%, RVD = 4.7%, ASD = 1.0 mm, RMSD = 1.8 mm, and MSD=19 mm were assigned with a score of 75. Therefore, a score of 75 meant the segmentation was as good as the manual delineation. The total score was the average of the scores for each validation measure. The scores received from evaluation are illustrated with a boxplot in Figure 13, where the average total score ± standard deviation is 83 ± 15, and all measures have an average value above 75. Moreover, in Table 3 the evaluation results are compared with the results of the previous semiautomatic methods for the SLIVER07 dataset. We can see the presented method receives a higher total score. Besides, our values of RVD and MSD are smaller as well. In particular, the MSD value is obviously lower than that of other methods. As we know, MSD would be large if there is an edge leakage and missing segmentation of discrete liver regions. The good thing is that our method can exactly avoid these problems. To be specific, for our method, region-growing is performed on the binary image, facing no risk of edge leakage, and with manual seed point setting, it can make sure to segment all discrete liver regions, avoiding missing segmentation. In addition, the unified LSM is resistant to edge leakage; coupled with the manual termination of the LSM in extreme cases, it can receive segmentation results of low MSD.

As illustrated in Table 2, for the validation results of the 3Dircadb dataset, the absolute values of all five measures are larger than those of the SLIVER07 dataset, which should be attributed to more pathological livers in the 3Dircadb dataset, making the segmentation task more difficult. In Table 4 the evaluation results are compared with the results of two previous automatic methods for the 3Dircadb dataset. The method proposed by Li et al. [44] was based on shape constraints and deformable graph cuts. In this method, a statistical shape model was constructed first based on the principal component analysis. Then the mean shape model was moved using thresholding and Euclidean distance transformation to obtain coarse segmentation and followed by a deformable graph cuts algorithm to yield final segmentation. The method of Erdt et al. [45] was based on learned shape priors with observed shape deviation. Specifically, their SSM was built on 220 nonpublic reference shapes of the liver, and the local shape variation was incorporated into the deformation term. With multi-tiered model adaptation, their method was able to receive expected segmentation results. From the results in Table 4 we can note that the two automatic methods could require less running time to process a CT volume, but they extra require time-consuming training process and statistical model construction, and their segmentation accuracy needs to be further improved.

**Figure 14:**
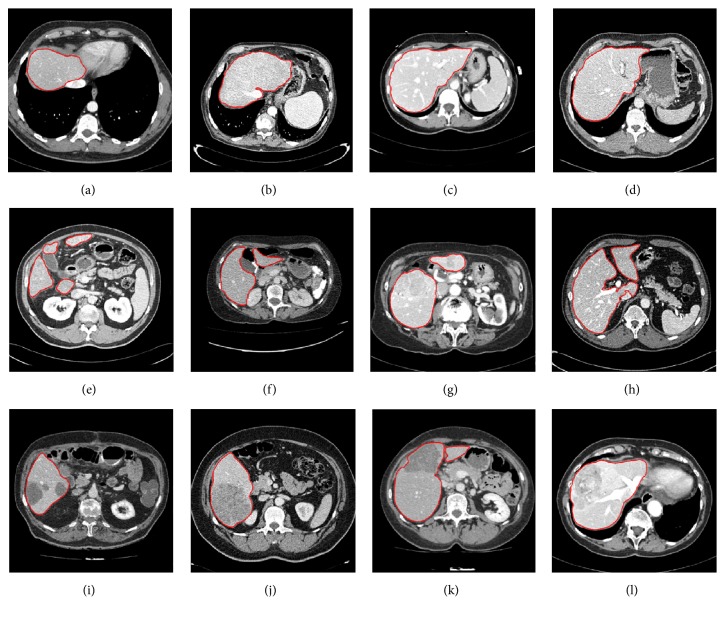
Some randomly selected liver segmentation results denoted in red: (a)-(d) indicate four simple examples of healthy livers having an indiscrete region; (e)-(h) indicate four examples of livers having discrete regions; (i)-(l) indicate four examples of pathological livers.

Some randomly selected liver segmentation results denoted in red are shown in Figure 14, where Figures 14(a)–14(d) denote four simple examples of healthy livers having an indiscrete region, Figures 14(e)–14(h) indicate four examples of livers having discrete regions, and Figures 14(i)–14(l) indicate four examples of pathological livers. From the segmentation results, it can be observed that our method is able to deal with most kinds of livers. For instance, there is almost no edge between the liver and the heart in Figure 14(a), in such an extreme case, the unified LSM was terminated manually to ensure segmentation accuracy. See Figure 14(e); there is a liver having four discrete regions. In this case, we set seed points within each region to ensure region-growing can segment all discrete liver regions, avoiding missing segmentation. Results in Figures 14(i)–14(l) prove that our method is also applicable and robust to pathological liver segmentation.

### 3.4. Liver Tumor Segmentation

#### 3.4.1. Method Training and Parameter Setting

Here we also give the values of the most important parameters of the proposed method for liver tumor segmentation. For the region-based LSM, we set *v* = 1, *δ* = 1 [33] in (23) and set the iterations to 100. For the HMRF-EM algorithm, we set both the iterations of MAP and iterations of EM to 15. To determine an optimal group of the balloon force and iterations *t* for the unified LSM, as a training procedure similar to liver segmentation method training, we trained this method on the local hospital data via VOE. The smaller VOE meant the better setting. Through method training, we set *ϑ* = 3 and iterations *t* = 200 in (34).

#### 3.4.2. Method Comparison

**Figure 15:**
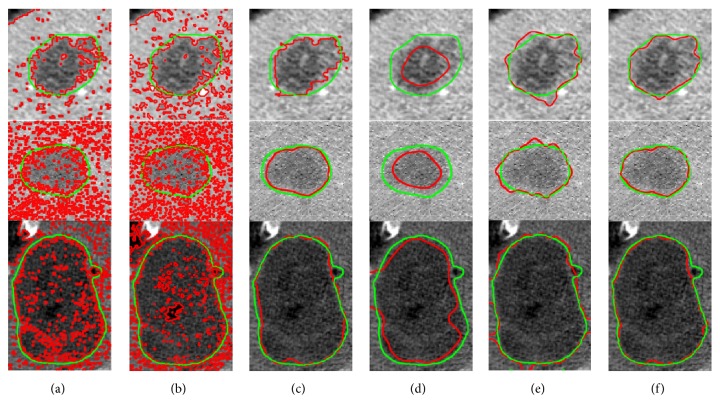
Comparison among our method and many other methods. The ground truth is denoted by green lines while the results obtained by different methods are in red. Columns from left to right indicate the results of, respectively, (a) the C-V model, (b) the HMRF-EM algorithm, (c) the region-DRLSE driven by the SPF, (d) the edge-DRLSE driven by the original *g*, (e) the edge-DRLSE driven by the enhanced edge indicator *g*_enhanced_, and (f) our LSM driven by both the SPF and the *g*_enhanced_.

In this section, to demonstrate the superiority of the unified LSM for liver tumor segmentation, we made a qualitative comparison of our method and many other methods. As illustrated in Figure 15, where three randomly selected ROIs are used, the ground truth is denoted by green lines while the results obtained by different methods are in red. Columns from left to right indicate the results of, respectively, the C-V model, the HMRF-EM algorithm, the region-DRLSE driven by the SPF, the edge-DRLSE driven by the original *g*, the edge-DRLSE driven by the enhanced edge indicator *g*_enhanced_, and our LSM driven by both the SPF and the *g*_enhanced_. Note that all methods were initialized with the same rectangle inside the tumor. From the results we can observe that our method performs best, receiving the closest segmentation to the ground truth. Specifically, due to the noise within the image, the C-V model and the HMRF-EM algorithm failed to work well, and they extra segmented nontumor tissues, requiring postprocessing such as removing disconnect regions and filling the holes to improve accuracy [49]. The original *g* was often vague, resulting in the worst segmentation. The *g*_enhanced_ provided more clear edge information than the original *g*, so the edge-DRLSE driven by the *g*_enhanced_ performed better than driven by the original *g*. The region-DRLSE driven by the SPF generally outperformed the C-V model and the HMRF-EM algorithm, but neither it nor the edge-DRLSE driven by the *g*_enhanced_ could achieve better segmentation than our LSM incorporating both the SPF and the *g*_enhanced_, indicating that combining more information could lead to more accurate segmentation.

#### 3.4.3. Validation Result

The proposed liver tumor segmentation method was validated with three datasets via five measures. Validation results are illustrated in Table 5.

For the hospital data, we built the ground truth with a radiologist from Ningbo Li Hui-Li Hospital, China. The hospital data includes 10 tumors, and most tumors have more obvious boundaries and higher contrast, tending to be easily segmented. Our method performed well on this dataset. The quantitative results for this training dataset were 15.52%, 9.02%, 1.87 mm, 2.5 mm, and 7.55 mm, respectively.

**Figure 16:**
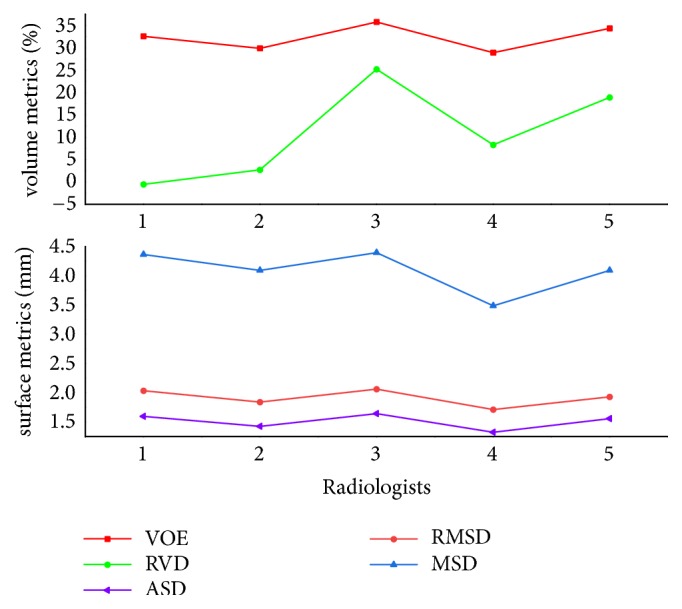
Five sets of validation results acquired by reference to corresponding manual delineations from five radiologists.

The MIDAS dataset provides manual segmentation from 5 radiologists, so it allows us to evaluate the robustness of our method according to different manual delineations. Note that we were only interested in metastasis tumors in this validation experiment, so the tumor from patient 4 was not used for method validation because it is a Hepatocellular carcinoma (HCC) [54]. For method validation, we compared each segmentation result with its five corresponding manual delineations to get five sets of metric values and took the average of the values for the five sets as the final metric values for each segmentation result. The quantitative validation results for this dataset are 32.19%, 10.07%, 1.51 mm, 1.92 mm, and 4.09 mm, as listed in Table 5, where we can notice that mean values of the two volume measures are relatively larger while mean values of the three surface measures stay smaller. The main reason for this could be that the liver tumors of the MIDAS data are small, even if a small segmentation deviation will lead to large values of volume measures. In addition, let us investigate the five sets of validation results acquired by reference to five corresponding manual delineations, as displayed in Figure 16, where we can note that the mean values of RVD in the five sets change over a wide range while the mean values of other four measures change relatively gently. On the one hand, this result explains that manual segmentation highly depends on the user experience, which introduces interobserver variability. On the other hand, all metric values are within a permissible range; namely, the inexistence of outliers proves that our method is robust to different manual delineations.

**Figure 17:**
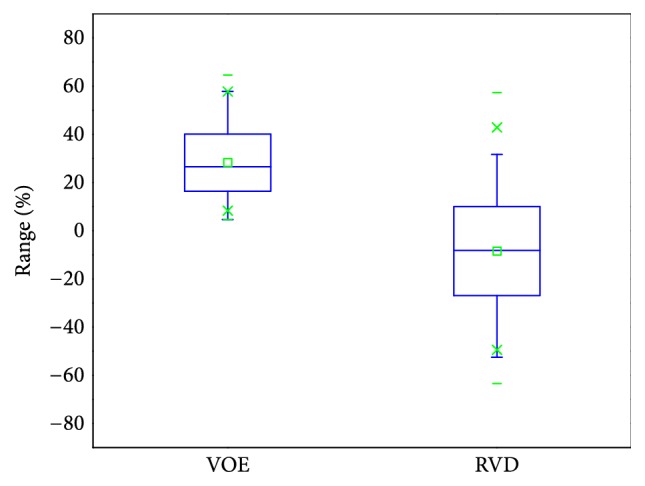
Results of volume metrics (VOE and RVD) for the 3Dircadb data, presented as a boxplot, where squares indicate the mean values.

**Figure 18:**
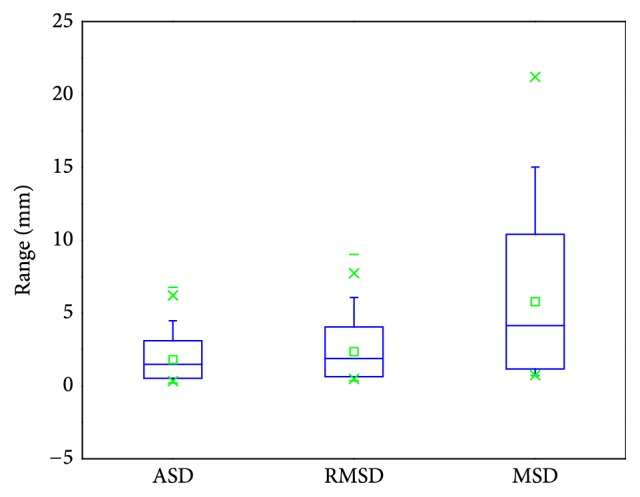
Results of surface metrics (ASD, RMSD, and MSD) for the 3Dircadb data, presented as a boxplot, where squares indicate the mean values.

We finally evaluated the proposed method with the public 3Dircadb data. This dataset provides 121 tumors and it is widely used for liver tumor segmentation method validation [14, 24, 46]. Values of five measures from validation were 28.22%, -8.46%, 1.81 mm, 2.35 mm, and 5.77 mm, respectively. The value of RVD was negative, indicating that the proposed method tends to obtain undersegmentation for this dataset. Besides, we present the results of volume measures and surface measures as boxplots, which are, respectively, illustrated in Figures 17 and 18, where green squares indicate the mean values. In addition, we compared our method with other state-of-the-art methods by analyzing the given results. As described in Table 6, it can be observed that the segmentation performance of our five metrics is superior to that of the method proposed by Foruzan et al. [46] and is comparable to the performance of Wu et al. [24]. Moreover, if we are only interested in volume metrics, we will find that our VOE value is slightly larger than that of the method presented by Moghbel et al. [14], and the absolute value of our RVD is very close to that of theirs. On the other hand, if we focus on surface distance metrics, we will observe that our ASD, RMSD, and MSD are smaller than that of Sun et al. [17] and Li et al. [30]. In addition, our method could not achieve the accuracy of the method introduced by Li et al. [47], but it avoids the massive training datasets and time-consuming training process required by their method. These above comparisons demonstrate that the performance of our method is comparable with that of the state-of-the-art methods.

**Figure 19:**
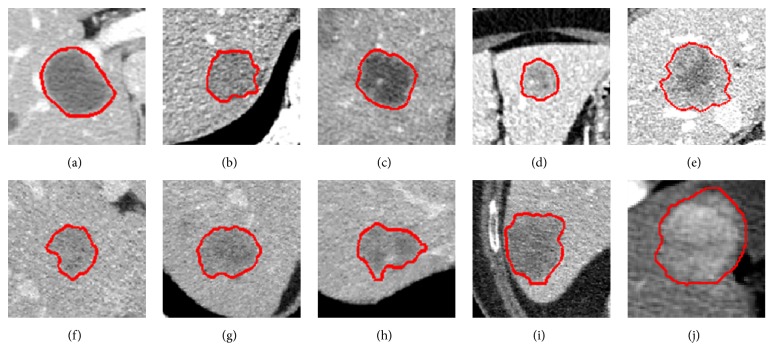
Some randomly selected liver tumor segmentation results denoted by red lines.

Some randomly selected liver tumor segmentation results denoted by red lines are presented in Figure 19. Among these typical liver tumors, we can observe that tumors in Figures 19(a) and 19(c) tend to be easily segmented due to more distinct boundaries. Tumors in Figures 19(b), 19(d), 19(e), and 19(j) have ambiguous edges. Tumors in Figures 19(f)–19(i) are characterized by low contrast. And tumors in Figures 19(d) and 19(e) are characterized by intensity inhomogeneities. These segmentation results show that our method could handle complex liver tumors well.

### 3.5. Interaction Time and Running Time

In this section, we discuss the interaction time and running time in our study.

#### 3.5.1. Interaction Time

**Figure 20:**
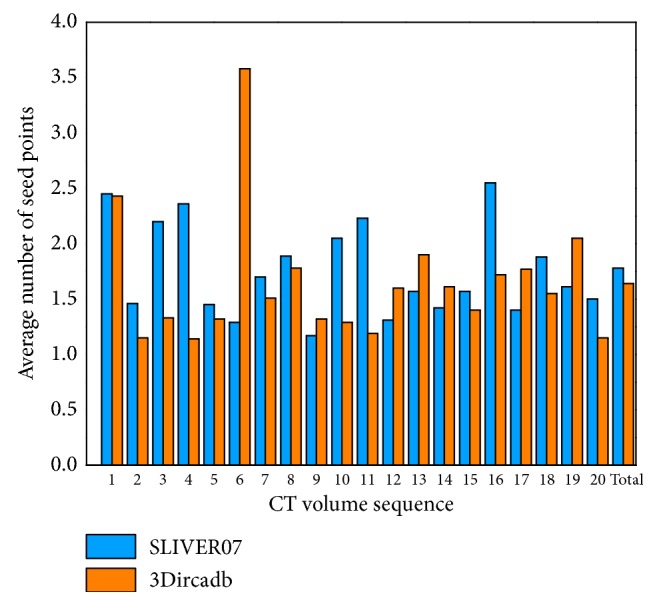
Bar chart: average number of seed points required for each CT image of all 40 CT volumes.

For liver segmentation, there are two parts to the interaction. On the one hand, the users are required to set seed points manually for region-growing. We calculated the average number of seed points needed for each CT image of all 40 CT volumes. As illustrated in Figure 20, where Total denotes the whole dataset, for the SLIVER07 dataset, most CT sequences require 1 to 2 seed points per image, and 1.8 ± 1.1 (average ± standard deviation) seed points are required per image for the whole dataset. For the 3Dircadb dataset, 1.6 ± 1.1 seed points are required per image. Moreover, we can note that the 6th CT sequence of the 3Dircadb dataset needs more than 3.5 seed points per image. This is mainly because there are more serious pathological changes in this volume. On the other hand, the users are asked to terminate the LSM evolution in some extreme cases to ensure segmentation accuracy, but this has no significant effect on the interaction burden since the proportion of extreme cases is small. The average total interaction time was about 86 s per CT volume according to statistics, so our interaction belongs to the medium interaction [2]. Besides, as illustrated in Table 3, the proposed method requires less interaction than other semiautomatic methods.

For liver tumor segmentation, the users are required to define the ROI and initialize the unified LSM manually. The average total interaction time was about 26 s per tumor according to statistics.

#### 3.5.2. Running Time

For liver segmentation, plus the interaction time, the average running time needed for each CT sequence was about 25 min, most of which was spent by the evolution of the unified LSM.

For liver tumor segmentation, plus the interaction time, the average running time needed was about 162 s per tumor. Specifically, time for the region-based LSM evolution only took up a small proportion while the HMRF-EM process and the unified LSM propagation spent the most running time. Running time of our method is modest, as illustrated in Table 6, where we can observe that our method is more efficient than the method proposed by Moghbel et al. [14] and is as efficient as the method of Foruzan et al. [46] but is less efficient than the method of Wu at al. [24].

### 3.6. Surface Rendering

**Figure 21:**
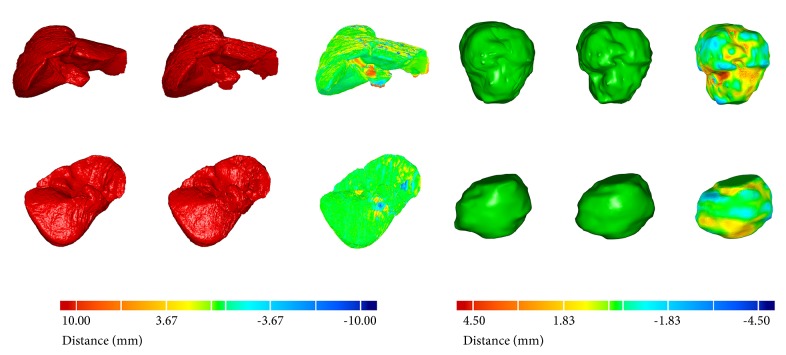
Two randomly selected surface renderings of liver and two randomly selected surface renderings of liver tumor. The left columns indicate the 3D visualizations of the ground truth, the middle columns indicate the 3D visualizations obtained via the proposed methods, and the right columns denote the surface distances between the first two, where red (positive value) indicates that the surfaces of the middle models are situated outside the surfaces of the left models and blue (negative value) inside.

Figure 21 shows two randomly selected surface renderings of liver and two randomly selected surface renderings of liver tumor. The left columns indicate the 3D visualizations of the ground truth, the middle columns indicate the 3D visualizations obtained via the proposed methods, and the right columns denote the surface distances between the first two, where red (positive value) indicates that the surfaces of the middle models are situated outside the surfaces of the left models and blue (negative value) inside. It can be observed that the segmentation results obtained by the proposed methods are very close to the manual delineations in 3D views. Specifically, the values of surface distances (ASD, RMSD, and MSD) of the livers in the first row and the second row were 0.18 mm, 1.77 mm, 9.6 mm and 0.02 mm, 1.34 mm, 8.50 mm, respectively; the values of ASD, RMSD, and MSD of the liver tumors in the first row and the second row were 0.23 mm, 0.68 mm, 4.51 mm and 0.20 mm, 0.77 mm, 2.13 mm, respectively. Accurate segmentation is the prerequisite for subsequent applications such as 3D real-time modeling of computer-aided surgery and 3D printing for artificial organs. Our methods are able to provide accurate and reliable segmentation for subsequent operations.

## 4. Discussion and Conclusion

In this paper, we have, respectively, presented a method for liver segmentation and a method for liver tumor segmentation. The two methods are grounded on a novel unified LSM, which is driven by both edge information and region information of image. In the following, we will discuss the advantages and shortcomings of the two methods, respectively.

The proposed liver segmentation method consists of a hybrid image preprocessing scheme, region-growing, and the unified LSM. The hybrid image preprocessing converts the input CT image into a binary image, providing a good condition for seed growth. In this way, threshold setting and seed initial location setting become simple and reliable. Moreover, region-growing requires fewer seed points to extract a rough liver region without risk of leakage. The unified LSM is applied at last for refinement to achieve optimal segmentation. Considering liver segmentation, three comparisons of our LSM and other two single-information driven LSMs have proved our LSM is able to adapt to a larger balloon force and more iterations, leading to more accurate segmentation results. The three comparisons are a qualitative comparison, a quantitative comparison, and a statistical comparison. From the result of qualitative comparison, we can observe visually that the proposed LSM is more resistant to edge leakage. In the quantitative comparison, VOE and RVD were used. From the result we can see that, with the increase in iterations, the curves of VOE and RVD belonging to the unified LSM are always under those belonging to other two LSMs and are more flat, indicating that the proposed LSM is able to yield more accurate results as well. The statistical comparison conducted at last makes the first two conclusions more convincing.

Method for liver segmentation was validated with two popular public datasets. Our method surpassed other previous semiautomatic methods on the SLIVER07 datasets, and all measures had a score above 75. Besides, the proposed method was compared with other two automatic methods on the 3Dircadb dataset. Though the two automatic methods were more time-efficient, the segmentation accuracy was lower than that of our method. Thus, we can conclude that the proposed method is competitive with both semiautomatic and automatic methods. Furthermore, from the validation results it can be obviously noted that the MSD value of our method is much lower than that of other methods. The ability of our method to bring lower MSD could be formed as follows: in our method, there is no risk of edge leakage when region-growing is performed on the binary image obtained through the hybrid image preprocessing. In addition, seed point setting incorporating prior information can make region-growing segment all discrete liver regions, avoiding missing segmentation. Moreover, the unified LSM can resist edge leakage; coupled with the manual termination of the LSM in extreme cases, it is able to receive segmentation results of lower MSD.

Our liver segmentation method requires less interaction than other semiautomatic methods. For instance, in our method, each CT image required 1.6 to 1.8 seed points, but in the method proposed by Yang et al. [19], 10 to 15 seed points were required for a large liver and 2 to 6 seed points for a small liver. So our method requires much fewer seed points. Besides, in the method of Chartrand et al. [21], the users were asked to initialize 3-10 contours manually first and perform manual postprocessing at last to improve accuracy; it took about 3 min for interaction per CT volume. In contrast, interaction spent about 86 s per CT volume in our study; it belongs to the medium interaction.

A limitation of the liver segmentation method is that running time has not been optimized, needing to be further reduced. During the segmentation process, the unified LSM propagation spent the most time. So for the future work, we will attempt to apply the narrowband scheme [25], the GPU acceleration scheme [55], or the coherent propagation algorithm [56] to boost level set evolution. Furthermore, inspired by the work of Cheng et al. [57] and Skalski et al. [58], we will consider adding the information of shape constraint and adjacent tissue constraint into the level set framework to further improve the accuracy of liver segmentation.

Liver tumor segmentation could be regarded as an optimization problem [31]. Due to the region information provided by the SPF, the unified LSM with an original edge indictor is able to tackle liver tumors with distinct edges and high contrast, but it is not competent to deal with complex tumors. Thus, in order to achieve an optimized solution, a local intensity clustering based LSM and the HMRF-EM scheme are employed to construct an enhanced edge indicator for the unified LSM. The local intensity clustering based LSM could handle intensity inhomogeneities well, and the HMRF-EM process has a strong low-contrast adaptability. So the unified LSM is able to tackle complex tumors that have blurred edges, low contrast, and intensity inhomogeneities. We made a qualitative comparison of our method and many other methods (the C-V model, the HMRF-EM algorithm, and other single-information driven LSMs). Our model performed best in this comparison, indicating the unified level set framework could receive optimized results for liver tumor segmentation.

The capabilities of the developed liver tumor segmentation method were evaluated with a varied collection of liver tumors. For the local hospital data, most tumors tend to be easily segmented because of obvious boundaries and high contrast. And we used this dataset for method training to determine optimal parameters. The MIDAS dataset provides manual segmentation from five radiologists. Validation with this dataset shows that our method is robust to different manual delineations. Our method was finally evaluated with the 3Dircadb dataset. The received measures were 28.2%, -8.5%, 1.8 mm, 2.4 mm, and 5.8 mm. The obtained RVD was negative, indicating our method tends to obtain undersegmentation for this dataset. Additionally, the comparison with other state-of-the-art methods on the 3Dircadb dataset shows the proposed method is competitive in both accuracy and efficiency.

Interaction required for the proposed tumor segmentation method is definition of the ROI and initialization of the unified LSM. Interaction time consumed was about 26 s per tumor in our study. Besides, the segmentation running time remains modest. It took about 162 s to segment a tumor, most of which was spent by the HMRF-EM algorithm and the unified LSM. The HMRF-EM algorithm is known to be a time-consuming algorithm, especially for handling large-size images [54]. So for the future work, in addition to accelerating the unified LSM evolution, we will consider proposing a scheme to boost the HMRF-EM process. Another limitation is that the unified LSM used for tumor segmentation needs to be initialized manually, though the initialization is simple that we define a rectangle inside the tumor as the initial contour. We will search for an approach for automatic initialization to further reduce the interaction burden. Last but not least, the extension of the methods to 3D would also be a major future goal.

In summary, we have, respectively, proposed a liver segmentation method and a liver tumor segmentation method. These two methods are mainly grounded on a novel double-information driven unified LSM, which could obtain more accurate segmentation. Validation with various datasets shows our methods provide accurate and reliable approaches for both liver and liver tumor segmentation. Our methods are competitive with previous methods in both accuracy and efficiency and can delineate boundaries that reach a level of accuracy comparable with those of human raters, which allows their adoptions in clinical practice.

## Figures and Tables

**Table 1:** 9 groups of the balloon force and iterations and corresponding RVD values.

Groups	RVD/%
*α* = 5, *t* = 50	-6.18
*α* = 5, *t* = 100	-2.02
*α* = 5, *t* = 150	0.85
*α* = 10, *t* = 50	-4.17
*α* = 10, *t* = 100	1.05
*α* = 10, *t* = 150	4.67
*α* = 15, *t* = 50	-2.69
*α* = 15, *t* = 100	3.37
*α* = 15, *t* = 150	8.34

**Table 2:** Segmentation performance of the proposed method on the two public datasets.

	VOE	RVD	ASD	RMSD	MSD
SLIVER07					
*Mean*	4.86	-0.70	0.90	1.59	7.82
*SD*	3.09	3.30	0.66	1.41	7.86
*Worst*	24.06	-18.03	4.51	8.63	49.90
*Best*	0.37	0	0.13	0.20	0.66
3Dircadb					
*Mean*	6.73	-1.02	1.29	2.04	9.68
*SD*	3.10	3.76	0.56	1.18	7.42
*Worst*	22.62	-18.33	3.65	6.95	45.03
*Best*	1.32	0	0.21	0.37	0.78

**Table 3:** Comparison of the proposed method with previous semiautomatic methods on the SLIVER07 dataset.

Method	Interaction	VOE	RVD	ASD	RMSD	MSD	Score
Beichel et al. [41]	High	5.2±0.9	1.0±1.7	0.8±0.2	1.4±0.4	15.7±3.5	82±2
Dawant et al. [42]	Medium	7.2±1.2	2.5±2.3	1.1±0.2	1.9±0.5	17.1±5.4	76±5
Beck et al. [43]	High	6.6±1.6	1.8±2.5	1.0±0.3	1.9±0.4	18.5±4.1	77±4
Chartrand et al. [21]	High	5.1±1.0	1.2±1.1	1.0±0.2	2.1±0.6	21.3±5.7	N/A
Eapen et al. [23]	N/A	7.3±0.8	1.3±0.5	1.1±1.0	1.7±0.4	15.7±2.6	78±2
Zareei et al. [22]	N/A	1.9±0.9	2.2±1.0	1.8±2.0	2.6±0.3	10.6±8.1	N/A
**Proposed**	**Medium**	**4.9**±**3.1**	**-0.7**±**3.3**	**0.9**±**0.7**	**1. 6**±**1.4**	** 7.8**±**7.9**	** 83**±**15**

**Table 4:** Comparison of the proposed method with previous automatic methods on the 3Dircadb dataset.

Method	Time	VOE	RVD	ASD	RMSD	MSD
Li et al. [44]	1.62 min	9.1±1.4	-0.1±3.6	1.6±0.4	3.2±1.0	28.2±8.3
Erdt et al. [45]	0.75 min	10.3±3.1	1.6±6.5	1.7±0.6	3.51±1.2	26.8±8.9
Proposed	24.6 min	6.7±3.1	-1.0±3.8	1.3±0.6	2.0±1.2	9.7±7.4

**Table 5:** Segmentation performance of the proposed liver tumor segmentation method on three datasets.

	VOE	RVD	ASD	RMSD	MSD
*Hospital data*					
Mean	15.52	9.02	1.87	2.50	7.55
SD	7.01	6.96	0.97	1.18	3.71
Worst	38.46	26.56	6.43	7.11	17.00
Best	3.91	0	0.54	0.73	1.41
*MIDAS*					
Mean	32.19	10.07	1.51	1.92	4.09
SD	11.13	28.00	0.41	0.45	1.43
Worst	86.96	93.55	2.97	3.85	10.94
Best	15.29	0	0.68	1.1	1.76
*3Dircadb*					
Mean	28.22	-8.46	1.81	2.35	5.77
SD	11.90	18.45	1.28	1.70	4.61
Worst	64.52	-63	6.77	9.03	25.64
Best	4.59	0	0.19	0.36	0.70

**Table 6:** Comparison of the proposed method with state-of-the-art methods on the 3Dircadb dataset.

Method	Mode	Time	VOE	RVD	ASD	RMSD	MSD
Moghbel et al. [14]	Auto	30s/slice	22.8±12.2	8.6±18.8	N/A	N/A	N/A
Sun et al. [17]	Auto	1s/slice	15.6±4.3	5.8±3.5	2.0±0.9	2.9±1.5	7.1±6.2
Wu et al. [24]	Semi	45s	29.0±8.2	-2.2±15.9	0.7±0.3	1.1±0.5	4.3±3.0
Li et al. [30]	Semi	N/A	14.4±5.3	8.1±2.1	2.4±0.8	2.9±0.7	7.2±3.1
Foruzan et al. [46]	Semi	154s	30.6±10.4	16.0±12.0	4.2±9.6	5.1±10.7	12.6±17.1
Li et al. [47]	Auto	30s-200s	11.7±4.3	-0.0±0.1	0.6±0.5	1.9±2.3	N/A
**Proposed**	**Semi**	**162s**	**28.2**±**11.9**	**-8.5**±**18.5**	**1.8**±**1.3**	**2.4**±**1.7**	**5.8**±**4.6**

## Data Availability

The data used to support the findings of this study are available from the corresponding author upon request.
